# Ventriculoperitoneal Shunt Alone for Cerebrospinal Fluid Rhinorrhea With Neuroendocrine Alterations in Idiopathic Intracranial Hypertension: A Case Report and Literature Review

**DOI:** 10.3389/fneur.2022.809224

**Published:** 2022-02-10

**Authors:** Deqing Peng, Kaichuang Yang, Cheng Wu, Faliang Gao, Weijun Sun, Gang Lu

**Affiliations:** Otolaryngology & Head and Neck Center, Department of Neurosurgery, Zhejiang Provincial People's Hospital, Affiliated People's Hospital, Hangzhou Medical College, Hangzhou, China

**Keywords:** idiopathic intracranial hypertension, CSF rhinorrhea, V-P shunt, neuroendocrine alterations, case report

## Abstract

Spontaneous skull base cerebrospinal fluid (CSF) leaks due to idiopathic intracranial hypertension (IIH) are a rare entity. Patients often present with CSF rhinorrhea, recurrent meningitis, chronic headache, and visual defects, while few patients have been reported to present with neuroendocrine alterations. Endonasal endoscopic repair is the first-line treatment for these leaks at present. However, the relatively high risk of recurrence remains the main cause of reoperation because of elevated intracranial pressure (ICP) after endoscopic surgery and absence of postoperative ICP management. A shunting procedure may stop CSF leakage or relieve symptoms in complex cases, and this is presently well-known as the last-line therapy for CSF liquorrhea. We describe a 29-year-old woman with spontaneous CSF rhinorrhea and neuroendocrine alterations due to IIH, and with no previous history of trauma, tumor, or nasal surgery. The bone defect in the skull base became implicated when the site of the leak was detected by cranial magnetic resonance imaging and computed tomography (CT). The patient was successfully managed *via* ventriculoperitoneal shunt (VPS) alone without endoscopic repair, and neuroendocrine alterations resolved after the shunting procedure.

## Introduction

Spontaneous cerebrospinal fluid (CSF) leaks due to idiopathic intracranial hypertension (IIH) are a relatively uncommon but serious disease. Patients generally manifest CSF liquor fistula, which leads to serious consequences such as meningitis, chronic headache, and visual defects, while few patients have been reported to present with neuroendocrine alterations (1). Endoscopic surgical repair is the first-line treatment performed when the diagnosis is confirmed. However, the relatively high risk of recurrence, which ranges from 25 to 87% (2), remains the main cause of reoperation because of elevated intracranial pressure (ICP) after endoscopic surgery and absence of postoperative ICP management. Some surgeons have attempted endoscopic endonasal repair, followed by a shunting procedure for high pressure, which may stop the CSF leakage or relieve the symptoms in complex cases (3). However, few surgeons have attempted to perform shunting procedures as the first-line therapy for CSF rhinorrhea.

This study presents an exceedingly rare clinical presentation of spontaneous CSF rhinorrhea, irregular menstruation, and infertility in an adult female patient who had elevated ICP. The patient was successfully managed *via* ventriculoperitoneal shunt (VPS) alone, without endoscopic endonasal skull base surgery.

## Case Presentation

A 29-year-old woman with a body mass index (BMI) of 35 kg/m^2^ who was previously healthy presented with complaints of intermittent watery discharge from the right nostril for 4 years when bowing her head, irregular menstruation, and infertility for 3 years. Her past medical and surgical history was unremarkable, and she had no history of head injury. Furthermore, no abnormalities were found in the physical examination, and no abnormalities were found during the ultrasound examination of the heart, liver, kidneys, uterus, or ovaries. Beta-2 transferrin was identified in the fluid outflow by biochemical analysis. Furthermore, the serum level of prolactin (PRL) was elevated (100 ng/ml). All other laboratory values for pituitary hormone were normal. No drugs were used to induce hyperprolactinemia, such as antiepileptic or antipsychotic drugs. The quantitative value of the ICP measured by lumbar puncture before treatment was 190 mmH_2_O.

**Figure 1:**
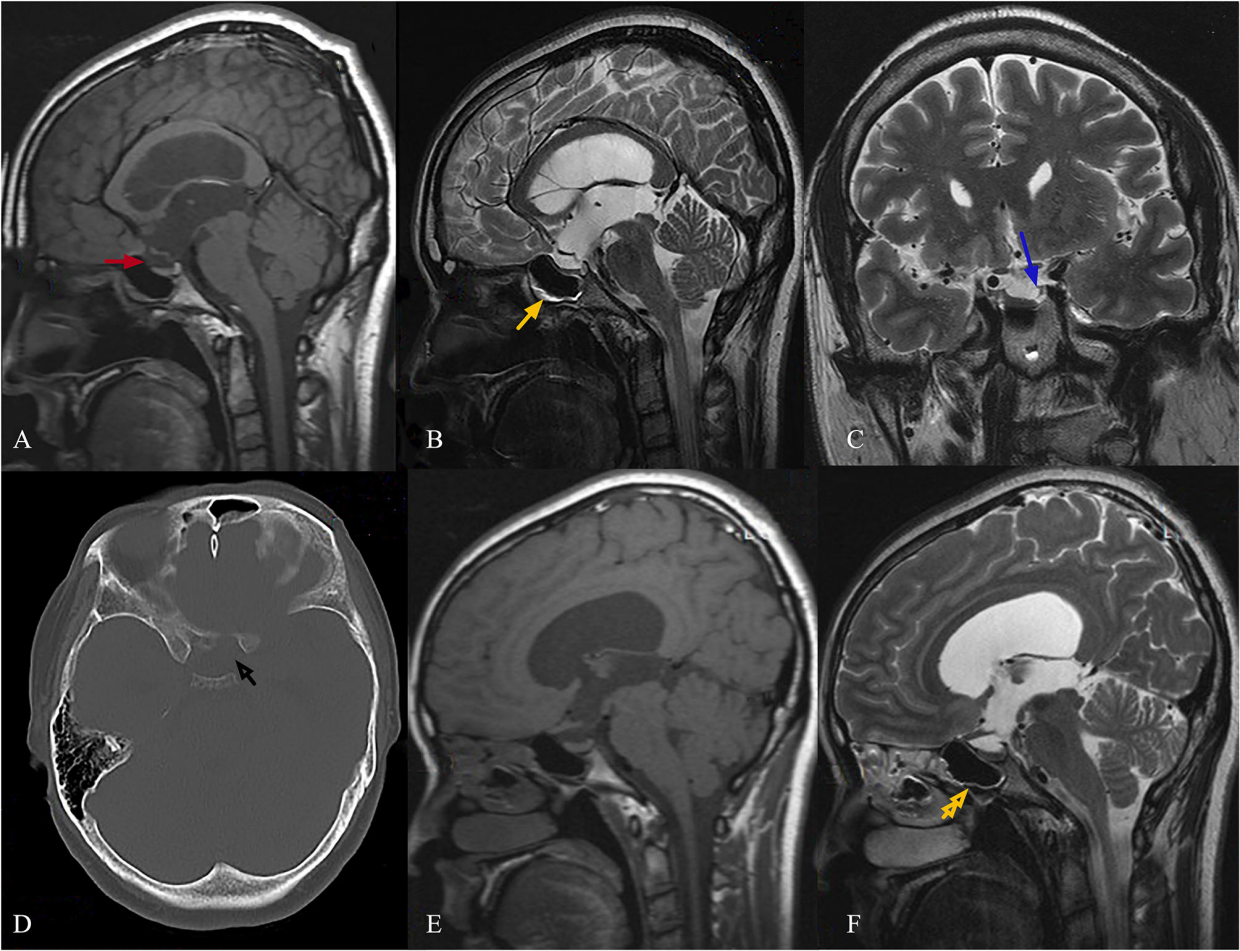
Female, 29-year-old, complained of runny nose for 4 years when bowing her head, and irregular menstruation with infertility for 3 years. The sagittal T1 images show a CSF-filled herniated sac through the bone defect into the sphenoidal sinus [**(A)**, red arrow]. The sagittal T2 images show that the fluid accumulated in the sphenoid sinus with a gas-liquid plane [**(B)**, yellow arrow]. A bone defect in the body of the sphenoid sinus, which was implicated at the site of the leak, was detected in an axial CT scan [**(D)**, black arrow]. The coronal T2 images show the presence of arachnoid pits along the anteromedial aspect [**(C)**, blue arrow]. Neurohypophysis was also noted, and this was compressed to the sellar floor, which became flat. The pituitary stalk was pulled long and thin **(A,B)**. At the 24th month of follow-up after the VPS procedure, the sagittal T2 images revealed no CSF accumulation in the sphenoid sinus [**(F)**, yellow double arrow]. The images also revealed change in pituitary stalk nodular thickening **(E,F)**.

The bone defect in the body of the sphenoid sinus implicated at the site of the leak was detected in the axial CT scan (Figure 1D). The coronal T2 images revealed the presence of arachnoid pits along the anteromedial aspect (Figure 1C). The sagittal T1 and T2 images revealed a CSF-filled herniated sac or bulge through the bone defect into the sphenoidal sinus (Figure 1A). There was no extended or embedded sac in the sphenoidal sinus, but there was a small amount of CSF (Figure 1B). The neurohypophysis was also noted to be compressed to the sellar floor, which became flat. The pituitary stalk was pulled long and thin. The magnetic resonance imaging (MRI) revealed obvious enlargement of the third ventricle, which was almost the same size as the lateral ventricles, and this was accompanied by slight enlargement.

Conservative therapy has been considered to have a low likelihood of success in stopping CSF leaks. In addition, the hyperprolactinemia caused by stretching of the stalk cannot be solved by endoscopic repair. Therefore, VPS procedure was recommended as the preferred treatment. The patient underwent VPS with the Medtronic device, without using acetazolamide, and the ICP value was regulated within 100–120 mmH_2_O. Then, the patient was followed up for 2 years after hospital discharge. The patient recovered well postoperatively with a normal PRL level in serum, had no clinical or radiological evidence of recurrence, and became pregnant twice. Furthermore, the sagittal T2 images revealed that no CSF accumulated in the sphenoid sinus (Figure 1F, yellow double arrow), and these imagines also revealed change in pituitary stalk nodular thickening at the 24th month of follow-up after VPS (Figures 1E,F).

## Discussion

Spontaneous CSF rhinorrhea is a unique clinical manifestation and has a strong association with presence of IIH. This disease is more common in middle-aged or older women. Relative to normal weight and underweight women, overweight and obese women have a greater risk, particularly when BMI is > 30 kg/m^2^ (4, 5). Patients often present with CSF rhinorrhea, recurrent meningitis, chronic headache, and visual defects, while rare cases of neuroendocrine alterations have been found in the literature (1).

Radiographic imaging and MRI features for spontaneous CSF rhinorrhea in IIH include the following three aspects: (1) findings of IIH, (2) bony defect(s) and herniating contents, and (3) potential risk factors, such as pterygoid recess pneumatization, arachnoid pits, or defects (6). To date, the most widely accepted explanation for the relationship between IIH and CSF leakage is that chronically rising ICP might be causative for the localized thinning of the bone in the skull base. Although there is controversy on the relationship between bone defect and CSF leakage, it was considered that bone defects occasionally occur (7).

The present case had imaging findings of IIH with enlarged lateral ventricles and third ventricle, and had evidence of elevated CSF pressure. The excellent CT scanning through the skull base identified bone defects in the body of the sphenoid sinus under optic chiasma, which was implicated at the site of CSF leak. The sagittal T2 images revealed that the fluid accumulated in the sphenoid sinus with a gas-liquid plane, and the coronal T2 images revealed the presence of arachnoid pits along the anteromedial aspect of the middle cranial fossa. No extended or embedded sac was found in the sphenoidal sinus.

Despite the conservative management with acetazolamide could have an effect on primary spontaneous CSF rhinorrhea (8), endonasal endoscopic repair still remains as the first-line or gold standard treatment for these leaks at present (9). Due to the potential mechanism of spontaneous CSF rhinorrhea in patients with IIH, it has been established that intermittent spontaneous CSF leaks through the arachnoid pits in the skull base dura was considered as a pressure release (1). When CSF leakage is closed by successful surgical repair, the ICP tends to increase because of blockage of CSF drainage into the nasal cavity, leading to long-term elevated ICP (2). Even though a successful surgical repair is completed, the unaddressed elevated ICP would contribute to the tendency of recurrence at the same or distant site (10). Emerging evidence supports that ICP management *via* medication or shunting procedure can be an important adjuvant treatment in this patient population (1). The reduction in ICP might reduce the rate of leak recurrence after nasal endoscopic repair in patients with evidence of IIH symptoms (9, 11). Furthermore, a recent study unveiled that postoperative permanent CSF diversion through LPS or VPS can decrease the recurrence rate by 11% (92.82 vs. 81.87%, *P* < 0.001) in patients undergoing surgical repair of CSF leakage (12).

Shunting is already well-known as the last-line therapy of liquor fistula for recurrent and refractory CSF leakage, especially for those who have failed a number of attempts of repair. The combination of VPS procedure and conventional endoscopic repair has been considered as a prospective approach (13). The shunting procedure was considered for the present case, even when endoscopic repair has not been carried out yet. It has been reported that the successful management of spontaneous CSF leak in this kind of patient is feasible by reducing ICP *via* the shunting procedure alone (14). The commonly held consensus is that LPS has a lower infection rate, and that this is possibly less invasive than VPS. However, a recent study revealed that LPS and VPS procedures have comparable rates of shunt failure and complications (15). The presented literature revealed that the LPS procedure is correlated with higher need for revision surgery (16). Therefore, the VPS procedure was chosen. To the best of our knowledge, it has not been reported that rare neuroendocrine alterations can be managed by VPS alone to date.

In this case, the patient presented with neuroendocrine alterations. Furthermore, the MRI revealed that the pituitary stalk was pulled long and thin, and that the pituitary was compressed to the sellar floor and became flat. The likely reason for this observation was the increase in ICP decreased intracranial compliance (17). In the meantime, hypopituitarism or hypoplasia would be induced by stretching of the pituitary stalk or optic nerves, because the high pressure in the sellar region cistern cannot be solved by repair. It was hypothesized that effectively reducing ICP maybe the key to its successful treatment. A VPS procedure without endoscopic endonasal repair changes the cerebrospinal fluid flow dynamics, and releases the pressure in the sellar region cistern. Decrease in ICP with the shunting procedure increases intracranial compliance, which could reduce the stretching or compression of the pituitary stalk or optic nerves and resolve neuroendocrine alterations. When the pressure of the CSF in the third ventricle is released, the CSF leakage through arachnoid pits in the skull base dura will also stop.

## Conclusion

The VPS procedure changes the CSF flow dynamics and releases the pressure in the sellar region cistern, increasing intracranial compliance. This may be a simple procedure to stop CSF leaks and relieve neuroendocrine alterations. The underlying mechanism and clinical implications of this finding remain unclear and could be of interest for further research.

## Data Availability Statement

The original contributions presented in the study are included in the article/Supplementary Material, further inquiries can be directed to the corresponding author/s.

## Ethics Statement

The studies involving human participants were reviewed and approved by the Institutional Review Board of Hangzhou Medical College Affiliated People's Hospital. The patients/participants provided their written informed consent to participate in this study. Written informed consent was obtained from the individual(s) for the publication of any potentially identifiable images or data included in this article.

## Author Contributions

GL contributed to the conception of the study. DP performed the investigation and data curation. KY modified the grammar mistakes. CW and WS contributed significantly to the analysis and manuscript preparation. FG helped to perform the analysis with constructive discussions. All authors contributed to the article and approved the submitted version.

## Funding

This work was supported by the Medical Health Science and Technology Project of Zhejiang Provincial Health Commission (2020372207).

## Conflict of Interest

The authors declare that the research was conducted in the absence of any commercial or financial relationships that could be construed as a potential conflict of interest.

## Publisher's Note

All claims expressed in this article are solely those of the authors and do not necessarily represent those of their affiliated organizations, or those of the publisher, the editors and the reviewers. Any product that may be evaluated in this article, or claim that may be made by its manufacturer, is not guaranteed or endorsed by the publisher.
